# Network-based features for retinal fundus vessel structure analysis

**DOI:** 10.1371/journal.pone.0220132

**Published:** 2019-07-25

**Authors:** Pablo Amil, Cesar F. Reyes-Manzano, Lev Guzmán-Vargas, Irene Sendiña-Nadal, Cristina Masoller

**Affiliations:** 1 Nonlinear Dynamics, Nonlinear Optics and Lasers, Universitat Politècnica de Catalunya, Terrassa, Spain; 2 Unidad Profesional Interdisciplinaria en Ingeniería y Tecnologías Avanzadas, Instituto Politécnico Nacional, Gustavo A. Madero, Ciudad de México, México; 3 Complex Systems Group & GISC, Universidad Rey Juan Carlos, Madrid, Spain; 4 Center for Biomedical Technology, Universidad Politécnica de Madrid, Madrid, Spain; Boston Medical Center, Boston University School of Medicine, UNITED STATES

## Abstract

Retinal fundus imaging is a non-invasive method that allows visualizing the structure of the blood vessels in the retina whose features may indicate the presence of diseases such as diabetic retinopathy (DR) and glaucoma. Here we present a novel method to analyze and quantify changes in the retinal blood vessel structure in patients diagnosed with glaucoma or with DR. First, we use an automatic unsupervised segmentation algorithm to extract a tree-like graph from the retina blood vessel structure. The nodes of the graph represent branching (bifurcation) points and endpoints, while the links represent vessel segments that connect the nodes. Then, we quantify structural differences between the graphs extracted from the groups of healthy and non-healthy patients. We also use fractal analysis to characterize the extracted graphs. Applying these techniques to three retina fundus image databases we find significant differences between the healthy and non-healthy groups (p-values lower than 0.005 or 0.001 depending on the method and on the database). The results are sensitive to the segmentation method (manual or automatic) and to the resolution of the images.

## Introduction

Fundus images are nowadays routinely used for the early diagnostic of ocular pathologies such as glaucoma [1–5] or diabetic retinopathy [6–14]. Other retinal imaging techniques are also used for manual and automatic diagnosis of these and other diseases [15]. Unsupervised algorithms can be used for automated retinal health screening, to differentiate normal fundus images from abnormal ones (age-related macular degeneration, diabetic retinopathy, and glaucoma) [16]. Studying the vascular structure of the retina can also advance our understanding of cardiovascular diseases [17, 18] and brain deceases, such as: Alzheimer [19] or dementia [20] due to changes in retinal microvasculature that may reflect similar changes in cerebral microvasculature. The performance of the analysis algorithms not only depends on the imaging technique and resolution [21] but also, on the methods used to segment the vessel network [22–30]. A main challenge for comparing the performance of different algorithms is that the performance of competitive algorithms is reaching the human intra-reader variability limit [10].

An analysis method with potential for diabetic retinopathy diagnosis is based on fractal analysis [31, 32]. While the fractal dimension of the blood vessels in the normal human retina is approximately 1.7 (consistent with a diffusion-limited growth process) [33], the fractal dimension of the vasculature tends to increase with the level of diabetic retinopathy [34]. However, the retinal fractal dimension varies considerably depending on the image quality, modality, and the technique used for measuring the fractal dimension [35]. The multifractal nature of the vascular network of the human retina [36, 37] and the reduction of the vasculature complexity with aging [38] have been reported. Fractal analysis has also been used to differentiate between healthy and pathological retinal texture [39].

Here we propose a new method that uses concepts inspired in network science [40–42]. We use the segmentation algorithm proposed in [43] to extract, from each digital fundus image, a tree-like graph where the nodes represent branching (bifurcation) points and endpoints, while the links represent vessel segments that connect two nodes. The graphs obtained are characterized by using the concept of node-distance distribution (NDD) [44], which is the fraction of nodes that are at distance *d* (shortest path) from a given node. We use as a reference node the optic disc (central node). To compare the extracted central distributions we use the Jensen-Shannon (JS) divergence that measures the distance between two probability distributions [45].

Precise graph comparison is a hard problem with many applications and different methods have been proposed in the literature (see [44, 46] and references therein). A main advantage of our approach is that it allows the comparison of graphs which have different numbers of nodes, and is appropriated for undirected and unlabelled graphs. Using a simpler metric (such as the Euclidean distance) can be more efficient for distinguishing different groups [47] but it only allows comparing graphs of the same size (i.e., with the same number of nodes), which is not the case for the graphs extracted from retina fundus images.

The proposed algorithm was tested on three databases of different size: a small high-resolution fundus (HRF) image database which comprises images of 15 patients with diabetic retinopathy, 15 with glaucoma and 15 without pathology; a large database, Messidor, where we used 230 images of patients with diabetic retinopathy classified in three groups, and 142 images of patients without pathology; and a medium size database from the Instituto de Microcirugía Ocular (IMO) which contains 70 images of glaucoma patients, and 23 images of patients without pathology. By means of nonlinear dimensionality reduction techniques we show that the DR, glaucoma and healthy groups, have statistically significant different features. To support these results, we also calculate the fractal dimension of the images (segmented and skeletonized versions) and find significant differences between the three groups, which are fully consistent with the results of the graph dissimilarity analysis.

## Methods

In this section we present the algorithms proposed for unsupervisedly retrieve features from images in a database. We also present the three databases we used to test our algorithms.

All the methods make use of the result of an unsupervised segmentation algorithm that was adapted from the one proposed in [43]. We start by filtering the photograph to enhance the contrast between the vessels and the background, and then we perform the Graph-based segmentation algorithm as proposed in [43], further details on the segmentation process can be found in the supporting information S1 Appendix. We refer to the raw segmentation result as a binary image whose pixels are 1 if they belong to a vessel and 0 if they belong to the background.

### Fractal dimension

The box counting algorithm is a well-known method for estimating the fractal dimension (FD) of a geometrical object [48]. It is based on the covering of an object with a grid of boxes of size *ε* and counting the number of boxes with information inside, *N*(*ε*). The dimension is an exponent that quantifies the scaling of *N*(*ε*) with the size *ε* as *ε* → 0, *N*(*ε*) ∼ *ε*^−*D*^, where D is the fractal dimension which can be cast as an equation:

$$\begin{array}{c} D=\lim_{\varepsilon \to 0}\frac{\log(N(\varepsilon ))}{\log(1/\varepsilon )} \end{array}$$ (1)

where *ε* → 0 is used to ensure coordinate invariance. We apply the box counting method to both, the raw segmented image (i.e., a binary image that includes all the pixels that correspond to vessels); and to the skeletonized image (i.e., a binary image where the width of each vessel segment was reduced to one pixel, without changing the length, location and orientation of each segment).

### Graphs extracted from segmented images

With the information retrived from the segmented images (raw and skeletonized) we construct undirected graphs where the nodes represent the branching (bifurcation) points and the endpoints, and the links represent vessel segments that connect pairs of nodes. The links have associated weights that represent the cost of transporting matter from one node to the other. If nodes i and j are not connected, *w*_*i*,*j*_ = 0, while if there is a segment connecting them, *w*_*i*,*j*_ ≠ 0. In order to test different possibilities using the values of the length, *L*_*i*,*j*_, and the width, *W*_*i*,*j*_, of the segment that connects nodes *i* and *j*, the weight of the link is defined as:

$$\begin{array}{c} w_{i,j}={\left(L_{i,j}\right)}^{l}{\left(W_{i,j}\right)}^{a} \end{array}$$ (2)

being *l* and *a* adjustable exponents, exploring, in this way, the group classification in terms of the length, the width and any product of powers of these two features. The length *L*_*i*,*j*_ and the width *W*_*i*,*j*_ of each link can be computed using the information contained in the skeletonized and raw segmentations. The length accounts for the number of pixels spanned by each link in the skeletonized version while the width can be estimated from the number of pixels (*N*_*i*,*j*_) each link has in the raw segmented mask as *N*_*i*,*j*_ = *L*_*i*,*j*_ × *W*_*i*,*j*_.

### Network measures

Structural differences between the extracted graphs were characterized by using the measures described in this section, which provide probability distribution functions (PDFs) that can be mutually compared by using nonlinear dimensionality reduction (NLDR) techniques.

#### Distributions of distances to the central node

The node distance distribution (NDD) measures the heterogeneity of a graph in terms of the connectivity distances, and allows the precise comparison of two graphs, by quantifying the differences between distance-based PDFs extracted from the graphs. It is based on computing, for each node *i*, the probability that another node *j* is connected to *i* with a path of distance *d*.

To apply the NDD concept to the tree-like graphs extracted from the segmented images, we consider only the distribution of distances to the central node that represents the optic nerve (because all the transported blood comes from and returns to this node). Thus, we analyze the Central NDD (C-NDD) PDF that gives the distribution of distances of the nodes to the central one. The distance of one node to the central one is defined as the sum of the weights of the shortest path.

**Fig 1:**
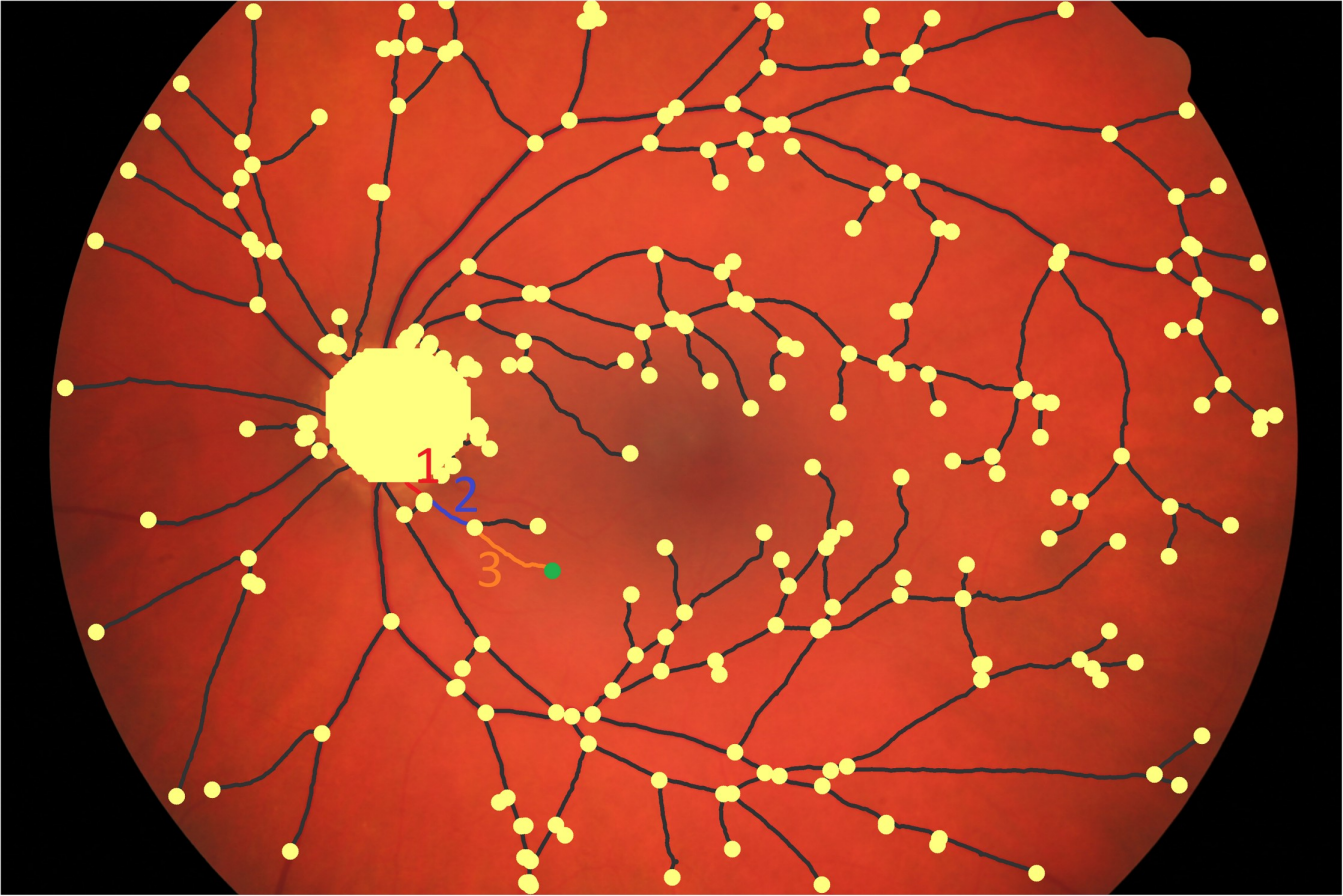
Segmentation example. Example result of the automatic segmentation algorithm on top of the original image, nodes are shown in light yellow, while links are shown in dark grey. An example of a shortest path from the optical disk to the node highlighted in green is shown, it consists of three links each one having its own weight according to Eq 2.

As an example, in Fig 1, the distance of the selected node to the central one is the sum of the weights of the three links that connect the two nodes. The distribution *P*_*CNDD*_ (*d*) is the fraction of nodes whose weighted distance from the central node is *d*, i.e. that the weighted shortest path of these nodes to the central node consists of links whose weights add up to *d*.

A variation of the Central NDD is the central mean weight distribution (CMWD), which is the distribution of average weights, i.e., the sum of the weights of the links that connect two nodes, divided by the number of links.

#### Weighted degree distribution

The degree distribution, *P*_*DD*_ (*k*), is a popular measure to describe the heterogeneity of the nodes of a graph. *P*_*DD*_ (*k*) is just the probability that a node has *k* links. In regular graphs all the nodes have the same number of links, and therefore, *P*_*DD*_ (*k*) is the delta-distribution, while in random graphs, *P*_*DD*_ (*k*) has a Gaussian-like shape. In weighted graphs, *P*_*WDD*_ (*s*) is the distribution of the strengths of the nodes (the strength, *s*, of a node *i* is the sum of the weights of its links, i.e., *s*_*i*_ = ∑_*j*_ *w*_*i*,*j*_).

#### Unsupervised nonlinear dimensionality reduction

The analyses described above provide us, for each image, with various probability distributions (one for each combination of *l*, *a*). These multidimensional descriptors carry several features which are often redundant. By using a nonlinear dimensionality reduction technique (NLDR), we are able to represent each distribution as a single point in a two-dimensional plane. In order to do that, we compare the distributions in a pair-wise manner using the Jensen-Shanon (JS) divergence [45]. In this way, we obtain a matrix, *P*, of dimension *N* × *N* (N being the total number of images analyzed) whose elements *p*_*i*,*j*_ are the distance (JS divergence) between the probability distributions extracted from images *i* and *j*. Then, using this matrix *P* as input for the IsoMap algorithm [49], it returns two features that are the coordinates of a point in a plane. This plane captures similarities and differences in the distributions such that similar distributions are represented as points close together and different distributions are represented as points far away from each other. It is worth noting that the algorithm is fully unsupervised, i.e. no prior image information (diagnosis) is needed at any step.

## Data

We used three different databases to test our algorithms.

### High-resolution fundus (HRF) image database

The HRF is a public database [50] (Download available at: https://www5.cs.fau.de/research/data/fundus-images/) which contains 45 color fundus images divided in 15 images of healthy patients, 15 images of patients with diabetic retinopathy and 15 images of glaucomatous patients. These images were captured using a Canon CR-1 fundus camera with a field of view of 60° and have a size of 3504 × 2336 pixels.

This database also includes a manual segmentation of the vessel network performed by a human expert. For comparison purposes we have also analyzed this set of images, as well as the images resulted from our automated segmentation method described in the supporting information.

### Messidor image database

This database, kindly provided by the Messidor program partners (see http://www.adcis.net/en/DownloadThirdParty/Messidor.html) consists of 1200 color fundus images taken with a field of view of 45º and resolutions ranging from 1440 × 960 pixels to 2304 × 1536 pixels [51]. Each image is categorized in one of four groups, corresponding to a diabetic patients without diabetic retinopathy and three increasing stages of diabetic retinopathy.

As our method is sensitive to changes in the images resolution, we worked with the first 400 images in the database that have a resolution of 2240 × 1488 pixels. Out of the 400 images we discarded 28 images in which the algorithm either failed to segment the network or to find the optic nerve and analyzed 372 images: 230 with diabetic retinopathy and 142 without.

### Instituto de Microcirugía Ocular (IMO) images

We also analyzed 93 images from patients at IMO (Ocular Microsurgery Institute: https://www.imo.es/en): 23 from healthy subjects and 70 from glaucoma patients. The images have a field of view of 45º and a resolution of 2000 × 1312 pixels. We used images from IMO database from consenting patients. The use of this data was approved by IMO’s Ethical committee for clinical study with date October 9th, 2018 (See S2 Appendix).

## Results

We applied the analysis tools described in Methods to the three retina fundus image databases. For the HRF database we performed the analysis using our automated segmentation and the manual segmentation provided with the database.

The algorithms were implemented in MatLab (segmentation, network retrieval, IsoMap) and Python (fractal analysis, network analysis) and their runtime using personal laptops was between 5 and 35 seconds per image depending on the resolution. These runtimes could be improved by rewriting the algorithms in a compiled language, however, they provide a rough assessment of the complexity of the algorithms.

We have summarized the results in two (Tables 1 and 2) where p-values were used to assess the statistical significance of the results obtained with the different methods. They were calculated with a t-test (using MatLab function ttest2) of the null hypothesis that the two samples come from distributions with equal means. For comparison, we also include references to other papers that have analyzed these databases and provided p-values.

**Table 1:** p-values comparing the healthy and diabetic groups.

Analysis	*l*	*a*	MESSIDOR p-Val.	HRF Automated p-Val.	HRF Manual p-Val.
C-NDD	1	-2	**0.011**	**0.0048**	**7.1e-05**
C-NDD	1	2	0.29	0.57	**6.4e-09**
CMWD	1	-2	0.82	0.68	**1.0e-10**
WDD	0	1	**0.0028**	**0.0070**	**8.0e-15**
Nodes	-	-	**0.0052**	0.074	0.69
Links	-	-	**0.0066**	0.073	0.99
Endpoints	-	-	**0.0054**	0.070	0.29
Bifurcation points	-	-	**0.0050**	0.082	0.65
FD skeletonized	-	-	0.23	0.88	0.68
FD raw	-	-	**1.3e-06**	**0.0096**	**0.0026**
FD best direction	-	-	**9.0e-12**	**4.5e-05**	**8.5e-06**
Best result using FD proposed in [35]	-	-	**0.01**	-	-
FD result in [52]	-	-	**0.0088**	-	-

p-values obtained by comparing the features extracted from the Messidor and HRF databases (automated and manual segmentations) of the groups with and without diabetic retinopathy (p-values smaller than 0.05 in **bold**).

**Table 2:** p-values comparing the healthy and Glaucoma groups.

Analysis	*l*	*a*	IMO p-Val.	HRF Automated p-Val.	HRF Manual p-Val.
C-NDD	1	-2	0.27	**0.0037**	**1.5e-06**
C-NDD	1	2	**5.8e-05**	**6.7e-05**	**0.00012**
CMWD	1	-2	**0.0066**	**0.00015**	**7.6e-07**
WDD	0	1	0.99	0.087	**1.1e-15**
Nodes	-	-	**0.012**	**0.0041**	**0.029**
Links	-	-	**0.012**	**0.0046**	**0.012**
Endpoints	-	-	**0.015**	**0.0042**	0.10
Bifurcation points	-	-	**0.0089**	**0.0034**	**0.026**
FD skeletonized	-	-	**0.0028**	**0.0038**	0.057
FD raw	-	-	0.11	**0.00058**	**0.0027**
FD best direction	-	-	**0.0015**	**0.00041**	**9.6e-08**
Best result in [53]	-	-	-	**<1e-6**	-

p-values obtained by comparing the features extracted from the IMO and HRF databases (automated and manual segmentations) of the healty and glaucoma groups (p-values smaller than 0.05 in **bold**).

### Fractal dimensions

**Fig 2:**
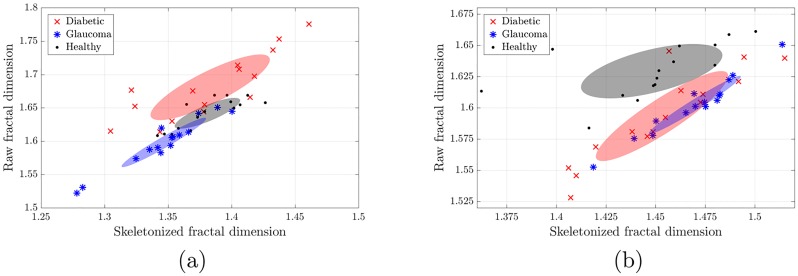
Fractal dimension analysis of the HRF database. Using the (a) automated and (b) manual segmentation. In both plots the horizontal axis denotes the fractal dimension of the skeletonized mask while the vertical axis accounts for the fractal dimension of the raw segmented mask. Each point represents the fractal dimensions of one image, while the ellipses represent the square root of the covariance matrix of each group. In (a) we note that the three groups are well separated (p-values 4.5e-05, 0.00041), while in (b) the healthy group is well separated from the non-healthy ones (p-values 8.5e-06, 9.6e-08). In both plots we note that using the two fractal dimensions improves the separation, in comparison to using only one.

Fig 2 displays, for the HRF database, the fractal dimension of the raw segmented images *vs*. the fractal dimension of the skeletonized ones for both the automated (panel a) and manual (panel b) segmentations. In the scatter plots each point corresponds to an image, while the ellipsoids represent the square root of the covariance matrix of each group (Diabetic, Glaucoma, and Healthy).

In both segmentations a clear distinction between healthy and non-healthy groups is obtained. In addition, with the automated segmentation a clear segregation between the three groups is obtained (left panel), which is not seen in the analysis of the manual segmentation (right panel).

Similar segregation between healthy and non-healthy groups is obtained for the Messidor and for the IMO databases, with p-values of the order of 9e-12 for Messidor (see Table 1) and 0.0015 for IMO (see Table 2).

### Central NDD

**Fig 3:**
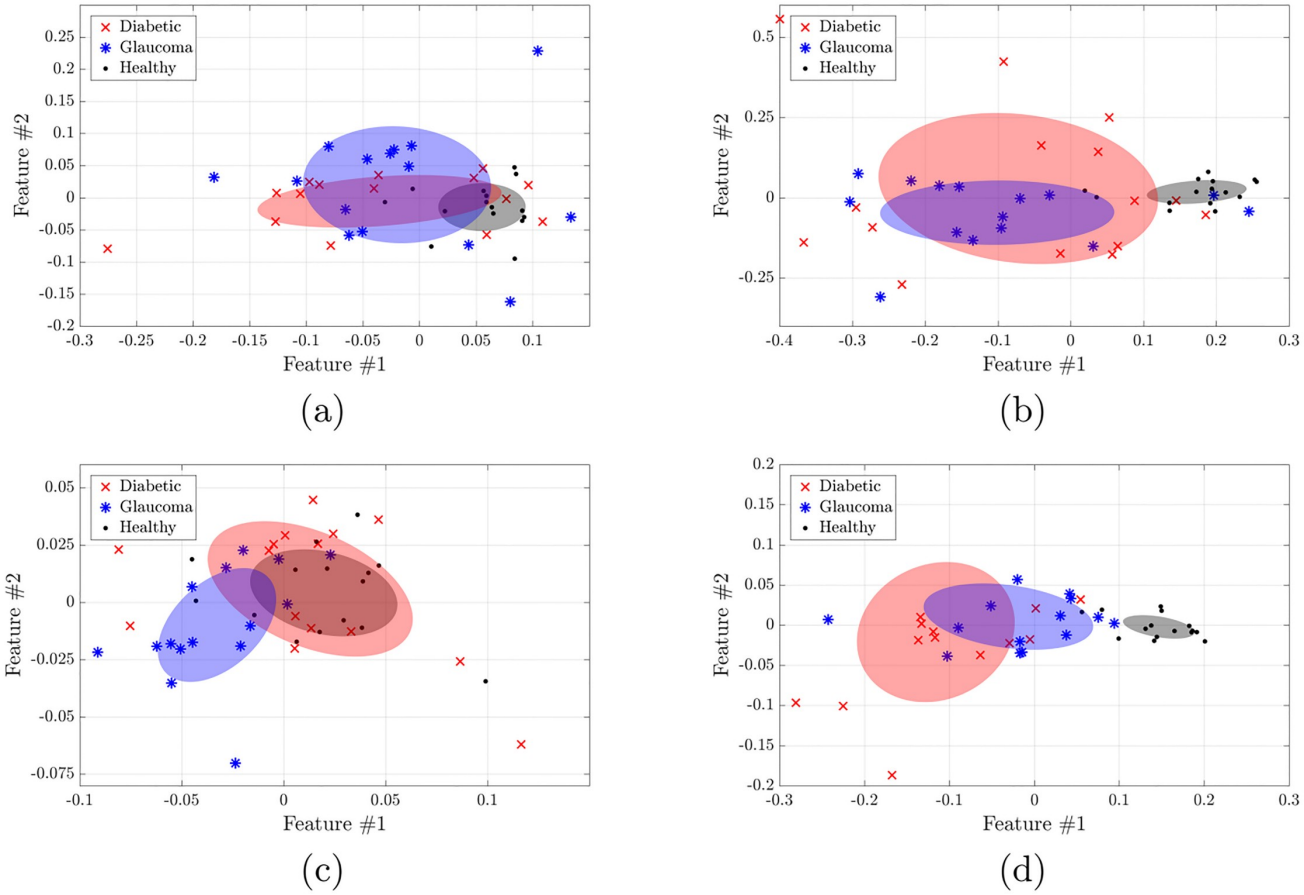
Central node distance distribution (C-NDD) and mean weight distribution (C-MWD) analysis of the HRF database. The panels display the IsoMap features extracted from the HRF database, using the automated (a,c) and manual (b,d) segmentations, with C-NDD analysis (a, b) and with C-MWD analysis (c,d). The weigths (Eq 2) are defined with *l* = 1 and *a* = −2. Here we note that both, C-NDD and C-MWD analyses perform very well in the manual segmentation (giving a clear distinction between the healthy and unhealthy groups), while in the automated segmentation they don’t provide a clear separation.

Fig 3(a) and 3(b). displays, for the HRF database, the results obtained from the C-NDD analysis with *l* = 1, *a* = −2 (since they provide the best differentiation between groups), for the automated (panel a), and manual (panel b) segmentations. As in Fig 2, here each point corresponds to the two features coming from the NLDR technique applied to the C-NDD histogram of each image and ellipsoids represent the square root of the covariance matrix for each group.

Again a clear distinction between the groups is obtained, with p-values (see Tables 1 and 2) in the order of 0.005 for automated segmentation and 1e-15 for manual segmentation.

### Central mean weight distribution

**Fig 4:**
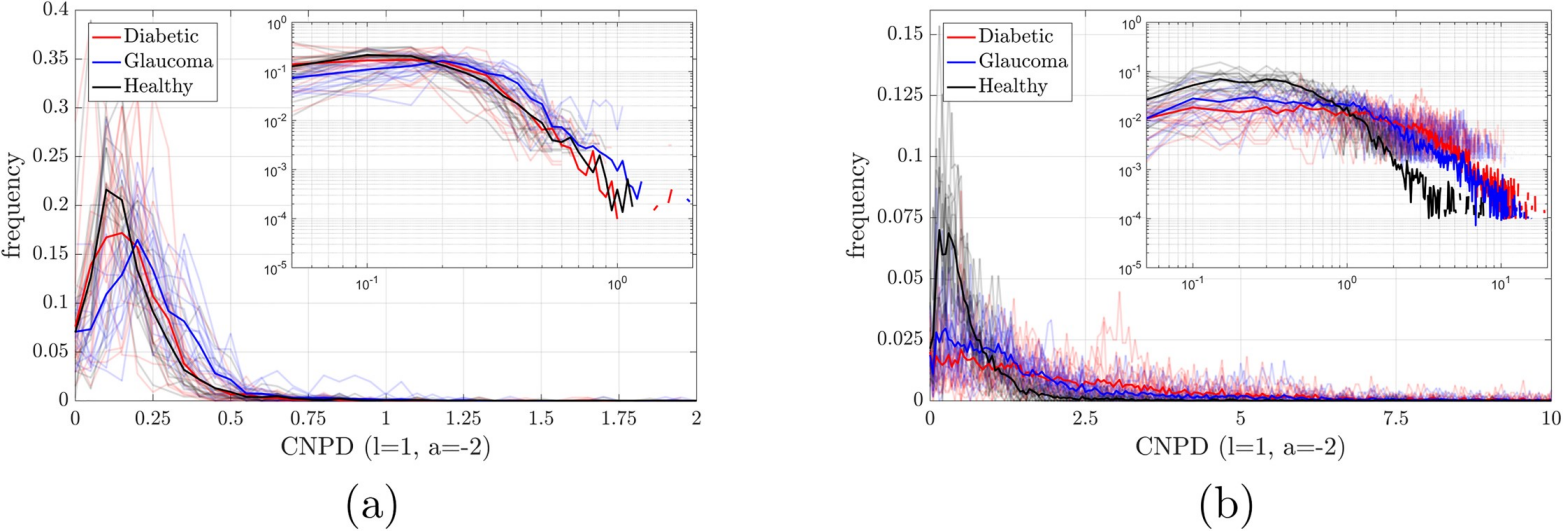
Central mean weight distribution (C-MWD) analysis of the HRF database. The panels display the raw histograms of the C-MWD extracted from the HRF database (with logarithmic scale in the insets), using the automated (a) and manual (b) segmentations (it corresponds to Fig 3(c) and 3(d)). The weights (Eq 2) are defined as *l* = 1 and *a* = −2. The mean histogram of each group is shown in a solid line, while every individual histogram was plotted with semi-transparent lines using the color corresponding to its group.

Fig 3(c) and 3(d). displays, for the HRF database, the results obtained from the CMWD analysis with *l* = 1, *a* = −2, using the automated (panel a), and manual (panel b) segmentations. Fig 4 shows the corresponding histograms. Here we see that for both segmentations, the distributions corresponding to healthy subjects tend to be more skewed to the left with respect to the pathological ones, however, as shown in Fig 3(c), the diabetic group and the normal group are indistinguishable using the automatic segmentation.

Similarly to the previous C-NDD analysis, in the manual segmentation the two non-healthy groups are clearly separated from the healthy one, while on the automated segmentation only the glaucoma group is found to be statistically different from the healthy one. The same results (not shown) hold for the Messidor and IMO databases.

Comparing C-NDD and C-MWD, one can see that the prior performs better for diabetic retinopathy while the latter performs better for glaucoma. The p-values are summarized in Tables 1 and 2.

### Weighted degree distribution

**Fig 5:**
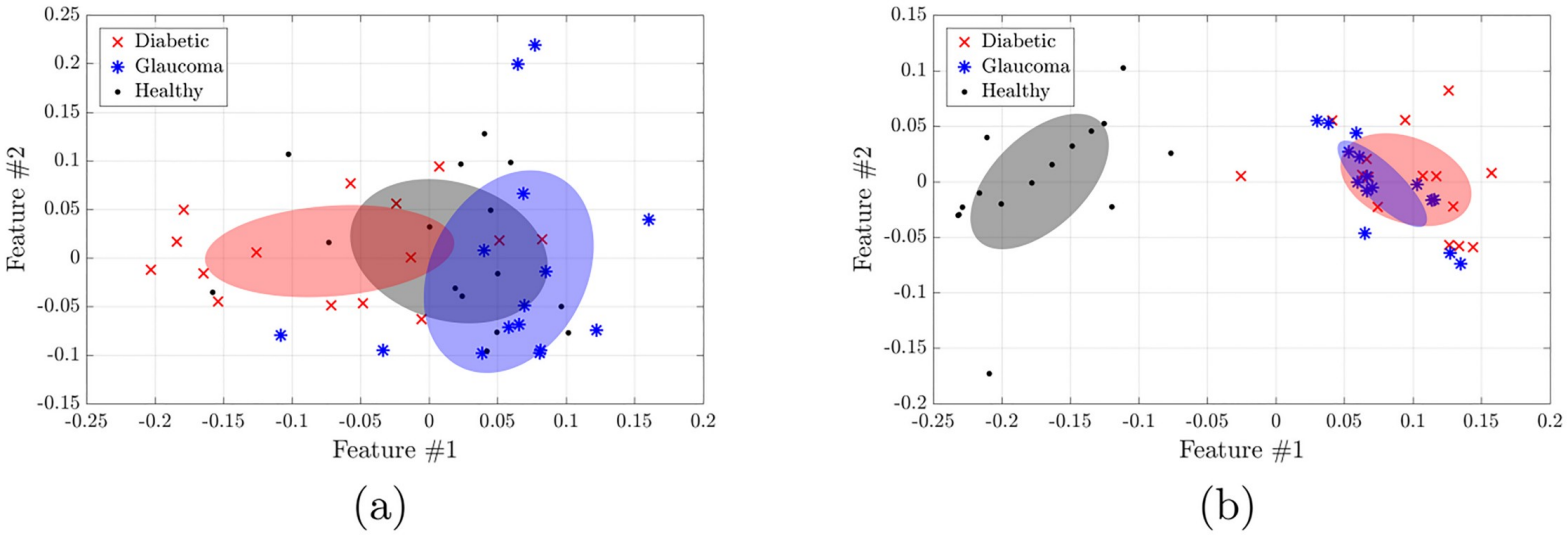
Weighted degree distribution (WDD) analysis of the HRF database. IsoMap features obtained from the (a) automated and (b) manual segmentations. The weigths (Eq 2) are defined with *l* = 0 and *a* = −1. We observe that with the manual segmentation the healthy group is clearly separated from the non-healty ones, while with the automated segmentation, the three groups are different, but they are not fully separated.

Fig 5 displays, for the HRF database, the results obtained from the WDD analysis with *l* = 0, *a* = 1 (i.e., the weight of the link just accounts for the vessel width), using the automated (panel a) and manual (panel b) segmentations.

Here we observe (as in the previous analysis, compare Fig 3) that in the manual segmentation the two non-healthy groups are quite separated from the healthy one. In the automated segmentation instead, only the diabetic group is statistically different from the healthy one. The same results hold for the Messidor database (see S1 Fig), while for the IMO database (not shown) the results are not significant. p-values are summarized in Tables 1 and 2.

### Analysis of other network features

We also analyzed other network features, such as the number of links, the number of nodes, the number of endpoints (nodes with only one neighbor) and the bifurcation points (nodes with 3 or more neighbors). The results, also presented in Tables 1 and 2, suggest that these basic features are not as informative as the features presented before: they have less significance (in terms of p-values) for identifying statistical differences between groups.

## Discussion

In Table 1 we show the results of the analysis of the diabetic and healthy groups from HRF and Messidor databases and in Table 2 the analysis of the Glaucoma and the healthy groups from HRF and IMO databases. In almost every case, the algorithms performed better when using the HRF manual segmentation compared to the automatic segmentation. We think that this is due to the intrinsic better quality of the manual segmentation, although it may also be attributed to observer bias. We don’t have a clear explanation of why, in a few cases, the automatic segmentation performs better than the manual one.

For the diabetic condition, in both Messidor and HRF with automated segmentation the best analysis turned out to be the best direction of the fractal dimension plane (i.e. a linear combination of both proposed fractal dimensions) while for the manual segmentation the best analysis was WDD with *l* = 0 and *a* = 1 (which was also the second best for Messidor), although all the proposed network based analysis were statistically significant. The methods that performed consistently well with both databases (and both segmentations) regarding diabetic retinopathy were C-NDD (*l* = 1, *a* = −2), WDD (*l* = 0, *a* = 1), the fractal dimension of the raw segmented image, and the best direction in the fractal dimension plane.

For the glaucoma case, in both IMO and HRF with automated segmentation the best results were obtained using the C-NDD with *l* = 1 and *a* = 2, while for the manual segmentation of HRF the best analysis was, again, WDD with *l* = 0 and *a* = 1, although all the proposed network based analysis were statistically significant. The methods that performed consistently good with both databases (and both segmentations) regarding glaucoma were C-NDD (*l* = 1, *a* = 2), CMWD (*l* = 1, *a* = −2), number of nodes, number of links, number of bifurcation points, and the best direction in the fractal dimension plane.

The parameters *l* and *a* chosen to test the network analysis have all a clear physical interpretation. The set *l* = 0 and *a* = 1 is simply the width of the corresponding vessel. The set *l* = 1 and *a* = 2 is proportional to the volume of such vessel. And finally, the set *l* = 1 and *a* = −2 can be related to the flow resistance of the vessel. Some other sets were tested such as the ones corresponding to the length and cross section of the vessel, obtaining no significant results.

It should be noted that the analyzed network is a 2-dimensional projection of the real 3-dimensional retina network, this implies that there are some nodes in it which, in reality, correspond to crossovers of veins and arteries. This alters the extracted features in two ways, by generating spurious nodes whose links are fictional, and by generating spurious shortest paths to the optical nerve. The problem of distinguishing arteries from veins in fundus photographies is highly non-trivial [54–56]. We have tested an algorithm based on prior work [57, 58] that eliminates spurious nodes (for example, those that have 4 links), but we found that the modification did not improve the performance of the proposed measures, while it added complexity and more parameters to the algorithm. We speculate that there are two reasons why the pruning of the spurious nodes does not improve the performance: 1) because most measures rely on the shortest path to the optical nerve, and only few paths are modified when comparing the “true network” with the 2D projected one, and 2) because the same kind of artifacts are present in all the images, thus, the comparison between images is still fair.

Our findings are consistent with the results recently reported in [59], where topological data analysis (TDA) was applied to the fundus images of diabetic retinopathy patients and healthy subjects in the HRF and MESSIDOR databases. The TDA features (that characterize connected components and holes in the images) allowed to discriminate between healthy patients and those with diabetic retinopathy in the HRF database but not in the MESSIDOR database, a fact that was interpreted as a due to the much lower resolution of MESSIDOR.

## Conclusion

We have demonstrated that the network-based features extracted from fundus images are useful for detecting topological changes produced in patients with diabetic retinopathy and glaucoma. For both diseases, the proposed network features we have proposed are able to separate the healthy group and the unhealthy groups with extremely high statistical significance. We have also compared our results with those obtained from fractal geometry analysis, and we have shown that using both fractal dimensions (raw segmented, and skeletonized) improves the separation between the groups, in comparison to using only one. The most statistically significant results were obtained using high resolution images (the HRF database), and in particular, when using the manual segmentation provided with the database. We found that analyzing the manual segmentation of the HRF database with the weighted degree distribution (see Fig 5) perfect classification could be achieved for both studied pathologies, and we note that this is not the case when using fractal analysis. In our study, it is apparent when comparing the results of manual and automated segmentation (in both diseases) that with the manual segmentation the classification performs almost always better than with the automated segmentation. Thus, improving the segmentation algorithm would probably improve the performance of the features derived from it. When analyzing images with lower resolution, the results show that the differences among the groups are not as statistically significant, and thus, we conclude that the topological differences found correspond to differences in the thinnest vessels of the network.

When analyzing diabetic patients, the weights that performed the best were the widths (*l* = 0 and *a* = −1 in Eq 2) and length/(width)^2^ (*l* = 1 and *a* = −2 in Eq 2). This can be understood by considering that diabetic retinopathy causes neovascularization that consists of thin vessels, and can also affect the vessel flow capacity. When analyzing glaucoma patients, the weight that performed the best was the volume (∝ length (width)^2^). This can be understood by considering that glaucoma is linked to an increase of the intraocular pressure, which can increase the volume of the vessels.

The measures proposed in this paper demonstrated very good performance in retina fundus images of different resolution, and of patients with different diseases. Therefore, it will be interesting to explore their potential with other vascular-related diseases.

## Supporting information

S1 Appendix Segmentation and network information retrieval. (PDF)

S2 Appendix IMO’s Ethical committee for clinical study approval. (PDF)

S1 Fig Extra figures. (PDF)

S1 Images Analyzed images from IMO. (RAR)
