# Supplementary material for: Network-based features for retinal fundus vessel structure analysis
Source: PLoS One. 2019 Jul 25;14(7):e0220132. doi: 10.1371/journal.pone.0220132 (PMC6658152; doi:10.1371/journal.pone.0220132)
Supplement: S1 Appendix — (PDF) [file pone.0220132.s001.pdf]

Supporting information

S1 Appendix. Segmentation and network information retrieval.

To retrieve the topological information of the vessel network in retinal fundus images the image processing workflow is divided in four steps, namely: preprocessing; segmentation; postprocessing; and network identification.

Preprocessing

In order to enhance the contrast between the vessel network and the retina, we apply a cascade of filters.

Each raw image (see Fig. 1A) is first equalized using a Contrast-limited Adaptive Histogram Equalization (Using MatLab function adapthisteq) separately to each color layer with 16-by-16 tiles and 256 bins.

Then, we perform a Gaussian high-pass filtering to retain the negative part alone of the resulting image. This way only the sudden drops of intensity are considered.

Next we apply a bank of 16 directional high-pass filters isotropically and compute the standard deviation pixel-by-pixel and color-by-color. This operation keeps only linear (one-dimensional) drops in the intensity of the original image (as vessels are) and disregards small points or areas (two-dimensional objects) with intensity drops.

The result is then masked with a simple mask computed by thresholding a blurred gray version of the original image.

An example of the resulting image after this preprocessing is shown in Fig. 1B.

Segmentation

For the segmentation we adapted the algorithm proposed in [3] to work with color images. We define the metric between colors in the CIE 1976 L*a*b* color space [2] that amplifies the differences in global intensities and attenuates the differences in the red channel intensities (as the differences between vessels and retina are mainly on the green channel and the red channel has more background variations unrelated to the vessel network). The outcome is depicted in Fig. 1C where the background appears in black and the foreground mask, containing the vessel network information, is shown in white. As a comparison, the corresponding manual segmentation performed by a human expert is shown in Fig. 1D.

We use a simplified version of the algorithm proposed in [1] for the optic disk localization to find and to segment the optical nerve, as it is shown in blue in Fig. 1E.

Postprocessing & network identification

After the image segmentation step a series of morphological operations is performed to further clean up the segmented image. This information is merged

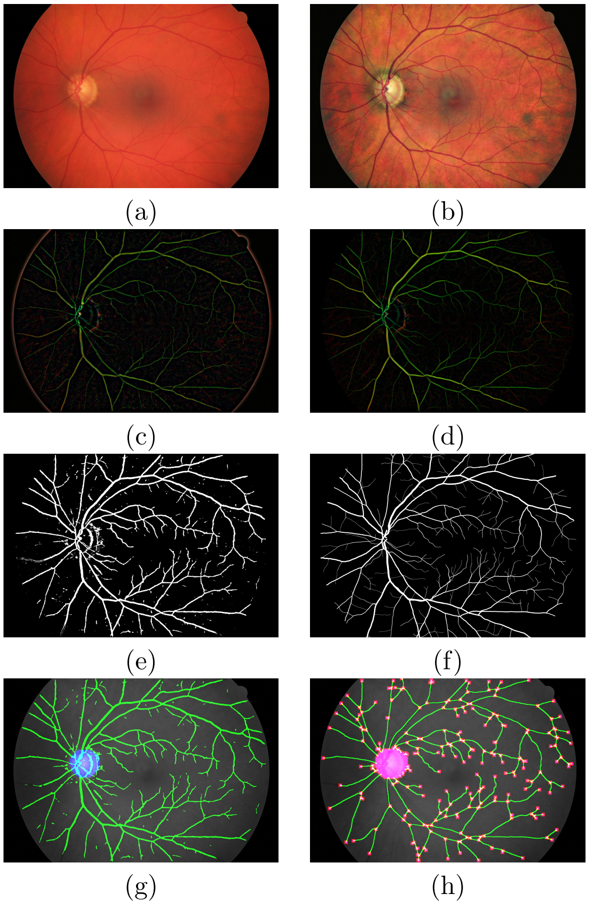

Figure 1: **Image processing workflow.** (a) Example image from the HRF database. (b) Image after the Contrast-limited Adaptive Histogram Equalization. (c) Gaussian high-pass filtering result. (d) Vessel enhancement result. Note that the arteries have a green color while veins have a slightly more yellowish color. (e) Raw segmentation result. (f) Manual segmentation performed by a human expert (for comparison). (g) Cleaned segmentation (in green), and optic nerve (in blue) superimposed to the gray-scale original image. (h) Network identification result, nodes in magenta and links in green superimposed to the gray-scale original image.

with the optical nerve as depicted in Fig. 1E. Finally, a skeletonization is performed (using the MatLab implementation in the bwmorph function) and the result is analyzed to retrieve the network structural information. The skeletonized version is topologically identical to the raw segmented mask, but the width of all vessels is set to one pixel (see Fig. 1F). Thus, the pixels that have one neighbor are endpoints (we identify them as nodes), the pixels that have

two neighbors are part of a vessel (we set them as part of a link connecting two nodes), and the pixels with three or more neighbors are bifurcation points (we identify them as nodes).

Therefore, we end up with a list of nodes (bifurcation points and endpoints) with their locations (marked in magenta in Fig. 1F), and the path of all the vessels connecting two nodes (colored in green). We define the whole optical nerve as a single node, allowing us to denote a central node and also disregard all possible vessel segmentation errors that may occur within the optical nerve. From the information plotted in Fig. 1F we can also retrieve an adjacency matrix, $A = (A_{i,j})$, by assigning each node a natural number (which are the indices of the matrix) and setting $A_{i,j} = 1$ if nodes i and j are connected with a link and $A_{i,j} = 0$ otherwise.The length $L_{i,j}$ and the width $W_{i,j}$ of each link can be computed using the information contained in the skeletonized and raw segmented masks. The length accounts for the number of pixels spanned by each link in the skeletonized version while the width can be estimated from the number of pixels ($N_{i,j}$) each link has in the raw segmented mask as $N_{i,j} = L_{i,j} \times W_{i,j}$.

References

[1] James Lowell, Andrew Hunter, David Steel, Ansu Basu, Robert Ryder, Eric Fletcher, and Lee Kennedy. Optic nerve head segmentation. *IEEE Transactions on Medical Imaging*, 23(2):256–264, 2004.

[2] K McLaren. Xiii-the development of the cie 1976 (l* a* b*) uniform colour space and colour-difference formula. *Journal of the Society of Dyers and Colourists*, 92(9):338–341, 1976.

[3] Daniel Santos-Sierra, Irene Sendiña-Nadal, Inmaculada Leyva, Juan A Almendral, Amir Ayali, Sarit Anava, Carmen Sánchez-Ávila, and Stefano Boccaletti. Graph-based unsupervised segmentation algorithm for cultured neuronal networks' structure characterization and modeling. *Cytometry Part A*, 87(6):513–523, 2015.
