# Supplementary material for: Network-based features for retinal fundus vessel structure analysis
Source: PLoS One. 2019 Jul 25;14(7):e0220132. doi: 10.1371/journal.pone.0220132 (PMC6658152; doi:10.1371/journal.pone.0220132)
Supplement: S2 Appendix — (PDF) [file pone.0220132.s002.pdf]

IMO
instituto de
microcirugía
ocular

DICTAMEN DEL COMITÉ ÉTICO DE INVESTIGACIÓN CLÍNICA

Don Rafael Navarro Alemany Presidente del Comité Ético de Investigación Clínica del **CEIC Institut de Microcirurgia Ocular - IMO**

CERTIFICA

Que este Comité, en su reunión de fecha 9 de Octubre 2018 (acta nº**159**/18), ha evaluado la propuesta del promotor relativa a:

Solicitud de evaluación del estudio:

COD IMO_181001_133
Probando métodos basados en redes complejas para el análisis y clasificación de fotografías de fondo de ojo. V1.0- 01/10/2018

IP Dr Jose L Güell IMO
CoIP Dra Cristina Masoller UPC , Pablo Amil, UPC, Fabián Reyes-Manzano UPC, Laura González IMO

Documentación presentada:
Solicitud de evaluación al CEIm del 3/10/2018, versión 1.0 de fecha 01/10/2018
CV resumidos de C Masoller PhD, Lic P Amil, Lic. C F Reyes

El ensayo ha sido evaluado por:

Dr. Rafael Navarro - Medicina - Presidente CEIm
Dña. Esther Canals- Enfermería-
Dña. Anna Mas- No sanitario- Pacientes
D.Leandro Martínez-Zurita- Jurista
Dña. Carolina Rovira – Farmacia AP
Dña. Pilar Sabin- Farmacia Hospitalaria
Dra Cecilia Salinas – Medicina
Dra Laia Pascual - Medicina

Este Comité, una vez evaluada la documentación presentada, CONSIDERA QUE:

El estudio clínico se plantea siguiendo los requisitos del:

- Orden SAS 3470/2009del 16 de Diciembre (estudios postautorización con medicamentos, y estudios observacionales)

Se cumplen los requisitos necesarios de idoneidad del protocolo en relación con los objetivos del estudio y es metodológica y éticamente correcto.

La capacidad del investigador y sus colaboradores y las instalaciones y medios disponibles son los apropiados para llevar a cabo el estudio.

Ronda de Dalt - Salida 7
Josep Maria Lladó, 3
08035 Barcelona
tel. +34 93 253 15 00
fax. +34 93 417 13 01
imo@imo.es / **www.imo.es**

IMO
instituto de
microcirugía
ocular

El Comité ACUERDA dar ***INFORME FAVORABLE***

Lo que firmo en Barcelona, a 11 de Octubre 2018

Firmado:
Dr Rafael Navarro
Presidente CEIC

Ronda de Dalt - Salida 7
Josep Maria Lladó, 3
08035 Barcelona
tel. +34 93 253 15 00
fax. +34 93 417 13 01
imo@imo.es / **www.imo.es**
