# Supplementary material for: Network-based features for retinal fundus vessel structure analysis
Source: PLoS One. 2019 Jul 25;14(7):e0220132. doi: 10.1371/journal.pone.0220132 (PMC6658152; doi:10.1371/journal.pone.0220132)
Supplement: S1 Fig — (PDF) [file pone.0220132.s003.pdf]

Supporting information

S2 Fig. Extra figures. Figures showing more examples of analyses made to the databases.

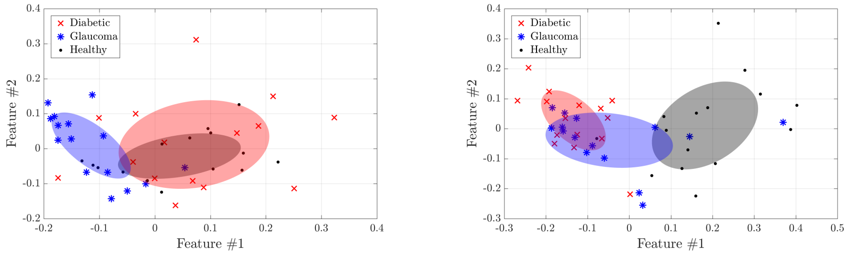

(a) (b)

Figure 1: **Central NDD analysis of the HRF database.** IsoMap features for the Central NDD analysis of the HRF database with $l = 1$ and $a = 2$ for the (a) automated and (b) manual segmentations.

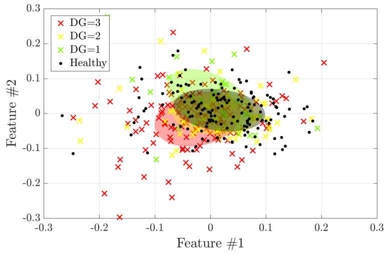

Figure 2: **WDD analysis of the Messidor database.** IsoMap features for the WDD analysis of the Messidor database with $l = 0$ and $a = 1$.

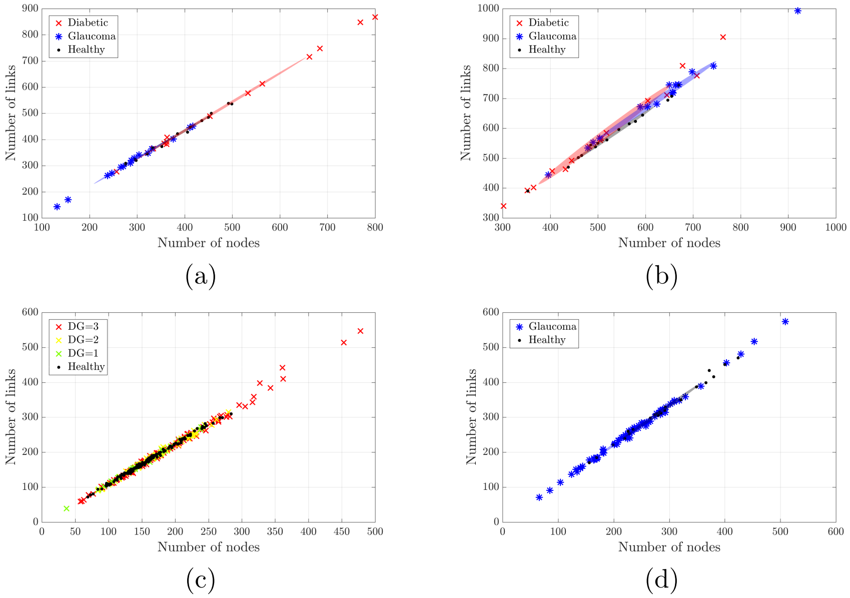

Figure 3: **Links *vs.* nodes.** Number of links *vs.* number of nodes for the HRF database using the automated (a) and manual (b) segmentations, and automatic segmentations of Messidor (c) and IMO (d) databases.
